# Potential Therapeutic Mechanisms and Tracking of Transplanted Stem Cells: Implications for Stroke Treatment

**DOI:** 10.1155/2017/2707082

**Published:** 2017-08-20

**Authors:** Yanhong Zhang, Honghong Yao

**Affiliations:** ^1^Department of Pharmacology, School of Medicine, Southeast University, Nanjing, Jiangsu 210009, China; ^2^Key Laboratory of Developmental Genes and Human Disease, Institute of Life Sciences, Southeast University, Nanjing, Jiangsu 210096, China

## Abstract

Stem cell therapy is a promising potential therapeutic strategy to treat cerebral ischemia in preclinical and clinical trials. Currently proposed treatments for stroke employing stem cells include the replacement of lost neurons and integration into the existing host circuitry, the release of growth factors to support and promote endogenous repair processes, and the secretion of extracellular vesicles containing proteins, noncoding RNA, or DNA to regulate gene expression in recipient cells and achieve immunomodulation. Progress has been made to elucidate the precise mechanisms underlying stem cell therapy and the homing, migration, distribution, and differentiation of transplanted stem cells *in vivo* using various imaging modalities. Noninvasive and safe tracer agents with high sensitivity and image resolution must be combined with long-term monitoring using imaging technology to determine the optimal therapy for stroke in terms of administration route, dosage, and timing. This review discusses potential therapeutic mechanisms of stem cell transplantation for the treatment of stroke and the limitations of current therapies. Methods to label transplanted cells and existing imaging systems for stem cell labeling and *in vivo* tracking will also be discussed.

## 1. Introduction

Stroke is a leading cause of death and long-term disability worldwide [1–5], and current epidemiological data suggest that the economic and social burdens of this disease will progressively increase over the next few decades. Approximately 795,000 individuals in the United States experience a stroke from 2003 to 2013 [6, 7]. Pathological subtypes comprise ischemic stroke and hemorrhagic stroke [8, 9]. In the Western world, ischemic stroke accounts for 87% of all stroke cases, and the remainder are hemorrhagic (intracerebral hemorrhage and subarachnoid hemorrhage) [6]. In ischemic stroke, an embolus or thrombus occludes a blood vessel, causing a reduction in blood blow to the brain and triggering a cascade of pathological responses associated with energy failure, excessive intracellular calcium, excessive excitatory amino acid release, the generation of reactive free oxygen species, and inflammation, ultimately causing irreversible brain impairment [10–12]. In the present study, numerous experiment animal models are used for the study of ischemic stroke, which are mainly divided into two broad categories: focal and global ischemia [13]. Focal ischemia is commonly used in basic research to mimic human stroke condition, which can be classified as transient or permanent occlusions. Among them, the middle cerebral artery occlusion (MCAO) model is widely accepted. Thread embolism is advanced through the external carotid artery to block the MCA resulting in consequent ischemic damage mainly in the corpus striatum and cortex brain regions [14].

To date, intravenous tissue plasminogen activator (tPA), which is only administered within 4.5 h of ischemic stroke, is effective [8, 15]. For patients who are unable to be treated within that therapeutic window, tPA is largely inadequate. Additionally, intravenous tPA enhances the risk of cerebral hemorrhage which limits its clinical application [16]. In recent year, another promising strategy for treatment of acute ischemic stroke is endovascular blood clot removal in large cerebral arteries with a stent retrieve [17, 18]. Numerous randomized trials have suggested that patients with a proximal cerebral arterial occlusion treated with rapid endovascular treatment could improve reperfusion and functional neurologic outcomes better than systemic tPA [19–21]. Numerous neuroprotective drugs targeting excitotoxicity, inflammation, or oxidative stress have proven unsuccessful [12, 22]. Conversely, emerging evidence indicates that stem cells may be a promising therapeutic avenue for cerebral ischemia. Stem cells possess self-renewal and multidirectional differentiation abilities [23]. At present, different types of stem cells are under investigation to determine their efficacy for the treatment of stroke, including mesenchymal stem cells (MSCs) [24], human umbilical cord blood mononuclear cells [25], neural stem cells (NSCs) [26], and adipose-derived progenitor cells [27]. Stem cell therapy has received considerable attention and is under extensive study, but the precise stem cell-mediated mechanisms governing improved outcomes after stroke remain unclear. Preclinical data suggest that stem cell therapy is a promising regenerative medical treatment given the limited capacity of the central nervous system (CNS) for self-repairs after ischemic stroke. Stem cells appear to release neurotrophic and growth factors to induce innate repair mechanisms, such as angiogenesis and neurogenesis [28, 29], in the adult brain and modulate the inflammatory response [30]. Additionally, stem cells secrete exosomes, which cross the blood-brain barrier (BBB) [31] to transfer certain proteins, noncoding RNA, and lipids to regulate recipient cells [32–34].

It is important to observe the survival, migration, distribution, and clearance of implanted stem cells to better understand their therapeutic mechanisms. *In vivo* imaging modalities for cell tracking are crucial tools for the development and optimization of stem cell therapy. Optical imaging, magnetic resonance imaging (MRI), magnetic particle imaging (MPI), and nuclear imaging, including single photon emission computerized tomography (SPECT) and positron emission tomography (PET), are generally used for cell tracking. Tracker agents must be safe, nontoxic, and biocompatible in clinical trials. Nanoparticles, particularly those labeled with superparamagnetic iron oxide (SPIO), are widely used in preclinical and clinical trials [35–37]. SPIO-labeled cells are tracked using MRI or MPI. SPECT and PET are used to track cells labeled with radioisotopes such as In-111-oxine [38] and ^125^iodine [39].

To further enhance the therapeutic effects of stem cells for the treatment of stroke and to determine an optimized therapeutic strategy, proper methods for cell labeling and appropriate imaging modalities must be employed. In this review, the potential therapeutic mechanisms of stem cell transplantation for the treatment of stroke and the limitations of current therapies will be discussed. We will also discuss methods for labeling transplanted cells and existing imaging systems for stem cell labeling and tracking *in vivo*.

## 2. Mechanisms of Stem Cell Transplantation to Treat Ischemic Stroke

### 2.1. Cell Replacement and Differentiation

Stem cell differentiation and appropriate incorporation into the existing neural network to replace the functions of lost neurons after transplantation represent critical aspects of cell-based therapy. Accumulating evidence suggests that transplanted stem cells have the ability to replace lost neurons via migration to damaged regions and promote neural differentiation, which contributes to behavioral improvements in different stroke models [40, 41]. Choi et al. [42] transplanted human bone marrow-derived mesenchymal stem cells (BM-MSCs) after photothrombotic ischemia and observed the elevated expression of neural and synaptic-relative proteins; additionally, the cells not only integrated well into the existing host circuitry but also enhanced endogenous neural differentiation in MSC-treated groups. At 7 days after transplantation, significant behavioral improvements appeared in the BM-MSC-treated group. Another study reported the utility of transplanting human embryonic stem cell- (hESC-) derived neural precursor cells (hNPCs) into the cortex to replace dying brain cells after permanent distal middle cerebral artery occlusion in rats, resulting in improved functional outcomes. The majority of transplanted hNPCs were positive for nestin, a marker of neural precursor cells. Approximately 10% of the cells differentiated into neuronal phenotypes 2 months after transplantation, and very few cells expressed astroglial or oligodendrocyte markers [43]. Other preclinical studies have reported the ability of NSCs from the human fetal striatum and cortex to survive, migrate, and differentiate into neurons in the stroke-damaged rat striatum [44]. Furthermore, homogenous populations of human neural stem cells (hNSCs) not only possess a remarkable ability to migrate into damaged regions and differentiate into neurons, astrocytes, and oligodendrocytes but also exhibit lower tumorigenicity *in vivo* [45]. Cheng et al. demonstrated the ability of intravenously delivered NSCs to traverse the BBB and migrate into the ischemic brain. Approximately 86% of transplanted NSCs maintained proliferative capability and enhanced the proliferation of endogenous cells. The intravenous administration of NSCs 24 h after stroke significantly improves functional deficits, but a reduction in cerebral infraction volume was not detected by TTC staining [46].

### 2.2. Endogenous Repair Mechanisms

Mounting evidence indicates that implanted stem cells accelerate long-term functional recovery by migrating toward the ischemic zone to enhance endogenous repair mechanisms via the secretion of growth factors [47, 48]. The adult mammalian brain contains a population of NSCs in the subventricular zone (SVZ) of the lateral ventricle that migrates to the olfactory bulb and generates new neurons [49, 50] and the subgranular zone (SGZ) of the hippocampal dentate gyrus (DG) [51, 52]. Brain injury, such as stroke, induces neurogenesis and angiogenesis [53–55] and promotes the proliferation and migration of neuroblasts or neural progenitor cells derived from the SVZ toward the injured site [56–58]. Angiogenesis is observed immediately after stroke because new blood vessels significantly increase by 3 days postinjury, and the proliferation of endothelial cells increases as early as 1 day postinjury [59]. Under ischemic conditions, SVZ multipotent NSCs derived from the stroke-injured cortex are capable of neurosphere formation and give rise to a subpopulation of reactive astrocytes in the cortex that contribute to astrogliosis and scar formation. Expression of the transcription factor *Ascl1* converts SVZ-derived reactive astrocytes into neurons *in vivo* [60]. However, the brain possesses a limited ability to form new neurons after injury, and endogenous regeneration mechanisms are insufficient to replace lost neurons [58]. Thus, there is a need to develop novel methods to enhance stroke-induced neurogenesis. Chromatin-modifying agents, which have previously been used as novel biological probes as well as for the treatment of cerebral ischemia, represent a viable method to stimulate endogenous NSCs and enhance NSC-mediated endogenous brain repair mechanisms [61]. Interestingly, channelrhodopsin-2 (ChR2) transgenic mice that undergo the optogenetic stimulation of glutamatergic activity in the striatum after stroke release glutamate into the SVZ, causing SVZ neuroblast proliferation and migration to the peri-infarct cortex via activation of the AMPA receptor. The stimulation of striatal glutamatergic activity may increase the survival and neuronal differentiation of recruited neuroblasts, thus improving functional recovery [62].

### 2.3. Secretion of Trophic Factors and Regulation of the Ischemic Microenvironment

Stem cells may regulate the neurovascular microenvironment to promote tissue repair and regeneration via autocrine or paracrine activity involving the release of cytokines, growth factors, or secreted extracellular vesicles. A recent study demonstrated the ability of extracellular vesicles released from stem cells to elicit biological functions similar to the stem cells themselves [63, 64], which represents a novel mechanism of intercellular communication, by delivering their cargo consisting of synaptic proteins, noncoding RNA, DNA, and lipids to acceptor cells, thus altering their gene expression under physiological and pathophysiological conditions [65–68]. Extracellular vesicles primarily include exosomes and microvesicles [69]. Exosomes are small (30–100 nm) membrane vesicles formed by the fusion of multivesicular bodies (MVBs) with the cell plasma membrane, are secreted by diverse cell types, and are present in body fluid such as blood, saliva, urine, and cerebrospinal fluid (CSF) [70, 71]. Exosomes are involved in cell communication, migration, angiogenesis, and cell growth processes in tumors and are considered natural carriers for applications in clinical trials. The systemic administration of exosomes released from mesenchymal stromal cells resulted in significant functional enhancement in the foot-fault test and a modified neurological severity score starting 2 weeks after treatment, as well as increased neurite remodeling, neurogenesis, and angiogenesis in the ischemic boundary zone after stroke in rats [72]. Further study demonstrated that exosomes harvested from microRNA 133b-overexpressing multipotent mesenchymal stromal cells improved neurological outcomes post-MCAO in rats beyond those elicited by naive exosomes because the exosomes indirectly downregulated the expression of Rab9 effector protein with kelch motifs (RABEPK) to further stimulate the release of exosomes from cultured primary astrocytes and then promote neurite outgrowth and elongation *in vitro* [73]. MRI suggested that the intravenous injection of xenogenic (from minipig) adipose-derived mesenchymal stem cells (ADMSC) and ADMSC-derived exosomes reduced brain infarct size 28 days after acute ischemic stroke, and neurological function underwent a significant improvement on day 14 following stroke. Moreover, in the xenogenic ADMSC/ADMSC-derived exosome treatment group, immune reactions and damage to major organs (brain, heart, lung, liver, and kidney) were not observed [74]. Exosomes generated from glioma stem cells promote the angiogenic capacity of endothelial cells by transferring miR-21 to downregulate the expression of vascular endothelial growth factor (VEGF) [32]. Stem cells enhance the endogenous repair capacity of the brain [32] and attenuate inflammatory reactions [75] though the secretion of trophic or growth factors. The majority of transplanted brain-derived neurotrophic factor- (BDNF-) overexpressing human NSCs express C-X-C chemokine receptor 4 (CXCR4), a chemokine receptor that is associated with inflammation [76]. Pretreatment of NSCs with BDNF causes the secretion of VEGF and macrophage colony-stimulating factor (M-CSF), CXCR4, and vascular cell adhesion molecule-1 (VCAM-1) expression and differentiation into mature neurons [77].

### 2.4. Alleviation of the Inflammatory Response

It is critical to alleviate the inflammatory response given its contribution to secondary brain injury after cerebral ischemia and experimental subarachnoid hemorrhage (eSAH) [78]. During cerebral ischemia, damaged tissue releases damage-associated molecular patterns (DAMPs) [79], which lead to a series of inflammatory responses such as the activation of microglia and the production of proinflammatory factors, followed by neutrophil recruitment and infiltration, which increase the permeability of the BBB [80, 81] and activate the complement system [82]. A variety of inflammatory factors regulate inflammation in the brain, such as tumor necrosis factor (TNF-*α*) and interleukin 1 (IL-1). MSCs possess the ability to orchestrate other cells to exert anti-inflammatory effects. Microglia cells incubated with IL-1-primed MSC conditioned medium increase their expression of anti-inflammatory, neurotrophic mediators and decrease their secretion of inflammatory markers such as interleukin 6 (IL-6), granulocyte colony-stimulating factor (G-CSF), and TNF-*α* [30]. MSCs and extracellular vesicles derived from MSCs intravenously injected after focal cerebral ischemia in mice were shown to modulate immune responses and attenuate postischemic immunosuppression in the peripheral blood [64]. Hypoxia-inducible factor 1-*α*- (Hif-1*α*-) modified MSCs implanted in a rat MCAO stroke model promote neurotrophin secretion while inhibiting the generation of proinflammatory cytokines [83]. According to Shichita et al., the efficient internalization of DAMPs, such as high-mobility-group box 1 (HMGB1), peroxiredoxins (PRXs), S100A8, and S100A9, is mediated by macrophage scavenger receptor 1 (MSR1) and macrophage receptor with collagenous structure (MARCO) in a murine model of ischemic stroke. Musculoaponeurotic fibrosarcoma bZIP transcription factor B (MAFB), a critical modulator of myeloid cell differentiation and proliferation [84, 85], enhances the expression of MSR1 in infiltrating myeloid cells. MSR1, MARCO, and MAFB deficiency causes the impaired clearance of DAMPs with consequent severe inflammation and neuronal injury [86].

### 2.5. Neuroprotective Effects and the Promotion of Axon Growth

Occlusion of a blood vessel by an embolus or thrombus causes a reduction in blood flow to the brain, which induces the disruption of the mitochondrial electron transport chain and the failure of oxidative phosphorylation. ATP supply fails, and excessive intracellular calcium is present in cells, ultimately causing neuronal damage [87]. Spermine and spermidine, which are free radical scavengers, have the ability to reduce lipid peroxidation [88] and modulate ion channels, receptor, and calcium trafficking [89]. In ischemic stroke, spermine significantly reduces infarction and neurological deficit [90]. Human mesenchymal stem cell treatment has a limited ability to restore cellular polyamine homeostasis, while levels of its metabolic products putrescine and spermidine significantly increase [91]. After CNS injuries such as ischemia and trauma, energy failure causing intracellular signaling disruption and several deleterious cascades are activated resulting in axonal degeneration and neuron death [92, 93]. In one systemic study, miR-133b overexpression in multipotent MSCs was systemically induced in rats subjected to MCAO. MiR-133b released from MSCs was transferred into astrocytes and neurons via exosomes both *in vitro* and *in vivo*, thus regulating connective tissue growth factor (CTGF) and *ras* homolog gene family member A (RhoA) expression and increasing axonal plasticity and neurite remodeling in the ischemic boundary zone (IBZ), subsequently promoting functional recovery after stroke [94, 95]. The transplantation of human neural progenitor cells 1 week after stroke significantly increases dendritic plasticity, promotes axonal rewiring, reduces the impairment of axonal transport, and enhances stem cell-induced functional recovery [47].

## 3. Limitations of Stem Cell Therapies

The clinical effectiveness of stem cell therapy is controversial, although accumulating evidence suggests that stem cell therapy has the potential to improve behavior and neurological function after experimental cerebral ischemia. Steinberg et al. [96] stereotactically implanted modified BM-MSCs into the brains of 18 patients with stroke. The surgical procedure and cell treatment were generally safe, and a significant improvement in neurological function was achieved after 12 months, which is consistent with a meta-analysis of preclinical studies indicating that stereotactic intracranial administration of MSCs significantly improves stroke outcomes [97]. Furthermore, human neuronal cells intracerebrally implanted into stroke patients with subcortical motor deficits measurably improved function in some patients, although a significant benefit in motor function was not observed [98]. A phase 2 trial comprising 58 patients with subacute ischemic stroke reported the safety of the intravenous administration of autologous bone marrow-derived mononuclear cells, but no beneficial improvements to neurological function were observed [99]. Another clinical trial suggested that the intra-arterial infusion of autologous bone marrow mononuclear stem cells results in minimal adverse reactions and may improve locomotion and language skills and decrease infarction volume, although these benefits were not significant compared with the nontreated group [100].

Stem cells represent an effective strategy to treat brain injury, but the precise mechanisms underlying stem cell therapy remain elusive due to the lack of appropriate cell tracking technology. Furthermore, the cell type, timing, dosage, and route of administration as well as the safety and biocompatibility of the tracker agents must all be considered. Stem cell therapy for the treatment of stroke improves functional recovery and offers the benefit of extending the intervention window via both intracerebral/intracranial (IC) transplantation and peripheral implantation routes, such as intravenous (IV), intra-arterial (IA), and intranasal administration [101]. IC transplantation is a more invasive procedure that allows precise injection into a chosen location, such as the penumbra and the ischemic core, to guarantee minimal cell delivery to untargeted areas [102, 103]. IV and IA systemic administration are less invasive and convenient approaches that results in the wide distribution of injected cells, but very low levels of cells migrate to the site of injury [38, 104]. Intranasal delivery of stem cells is noninvasive and targets the brain [105, 106]. Different administration routes cause the differential biodistribution of transplanted cells, although all routes improve functional recovery of the brain. Thus, it is critical to understand the homing of transplanted stem cells to sites of injury and to monitor transplant dynamic processes, including cell proliferation, migration, and biodistribution. To obtain optimal therapeutic effects and enhance our understanding of the mechanism by which stem cells promote functional recovery in neurological disorders, it is essential to develop noninvasive, reproducible, and quantitative *in vivo* imaging approaches to track stem cell fate. In recent year, the methods for *in vivo* labeling and tracking of implanted stem cells consist of MRI [39, 107], optical imaging (fluorescence and bioluminescence imaging) [108, 109], and nuclear imaging including SPECT [110] and PET [111].

## 4. *In Vivo* Imaging Systems and Tracker Agents for Transplanted Stem Cells

### 4.1. SPIO Nanoparticles

Extensive work has been done to synthesize and make surface modifications to SPIOs. Iron oxide nanoparticles are roughly divided into SPIO, ultrasmall SPIO (USPIO), monocrystalline iron oxide nanoparticles (MION), and micron-sized superparamagnetic iron oxide (MPIOs) based on size. SPIO contrast agents are particles composed of an iron-oxide core coated with dextran (ferumoxide) or carboxydextran (ferucarbotran) [112] and protamine sulfate (Pro), which are FDA-approved agents. SPIO nanoparticles are capable of labeling the vast majority of mammalian cells and are imageable during animal experiments and clinical trials. MRI is used to determine the homing, migration, and differentiation of stem cells labeled with SPIO [113, 114]. This image modality possesses high spatial resolution, which facilitates long-term and single-cell detection, and is noninvasive and utilizes nonionizing radiation. Cells labeled with SPIO exhibit low-intensity signals during T2 and T2^∗^ MRI imaging [113, 115]. MION labels stem cells without requiring the use of a transfection agent [116] and does not affect cell viability, phenotype, and *in vitro* differentiation capacity [112]. Many measures have been taken to improve labeling efficiency and enhance MRI detection sensitivity. Compounding fluorescent mesoporous silica-coated SPIO for stem cell MRI is used to enhance the detection sensitivity and efficiency for cell labeling with no adverse reactions [117, 118]. It is also useful to combine MRI with other noninvasive imaging modalities such as reporter gene-based molecular techniques to overcome any deficiencies and obtain more information on the behavior of implanted cells. hNSCs stably expressing enhanced green fluorescence protein (eGFP) and firefly luciferase (fLuc) reporter genes were labeled with SPIO for MRI and grafted into an experimental stroke model. The survival, tumorigenicity, and immunogenicity of grafted cells were efficiently tracked in real time and investigated for 2 months using multimodal MRI and bioluminescence imaging (BLI) techniques [41]. MSCs labeled with SPIO synthesized in the laboratory were intra-arterially injected in a canine stroke model, given its similarity to the human brain, and were tracked using *in vivo* 3.0 T MRI imaging for at least four weeks [119]. SPIO (448 *μ*g/mL) had no adverse effects on the viability of adipose-derived canine MSCs [120]. However, exact cell quantification using an MRI imaging system may result in errors because MRI possesses large background signals from subject interfaces, and certain pathological conditions such as hemorrhage cause similar MRI signals, resulting in mistakes during the measurement of iron-containing contrast agent accumulation.

Magnetic particle imaging (MPI) is a novel molecular imaging technique that is limited to magnetic tracers and directly images SPIO nanoparticle-tagged cells [121, 122]. SPIO tracers introduced into the body generate MPI signals, while animals themselves neither generate nor reduce MPI signals [123, 124]. Thus, MPI provides accurate quantification, high image contrast, and longitudinal observation to monitor the distribution and location of stem cells. MPI is very suitable for preclinical and clinical applications to evaluate functional brain physiology during pulmonary perfusion [125] and traumatic brain injury [126], and there are few background tissue signals using optimized long-circulation SPIO trackers [127]. MPI is applicable to track transplanted cell redistribution and localization *in vivo*. In a recent study, the intravenous administration and dynamic distribution of SPIO-labeled MSCs in rats were monitored using MPI. Tracer clearance from the body can also be quantified using longitudinal MPI [128]. In other studies, MPI is able to track the long-term fate of exogenously labeled human stem cells with high image contrast in the murine brain and whole body for weeks to months [129].

### 4.2. Radiopharmaceuticals

Cells labeled with radioisotopes are generally tracked more accurately using SPECT and PET given their extraordinary sensitivity and tissue penetration, minimal background signals, and capacity to scan an entire body to investigate cell distribution to other organs. Radiotracers lacking toxicity and effects on cell viability are urgently needed. ^111^In causes damage to labeled cells due to its radioactivity and toxicity, although it has a half-life of 67 h, thus allowing long-term monitoring of up to 14 days [130, 131]. Cells labeled with indium-111-oxine (^111^In oxine) exert low negative effects on cell viability [38]. However, the radioactive decay of usable tracers is not suitable for long-term tracking and limits the development of nuclear medicine techniques. Radioactive technetium-99m (^99m^Tc) and ^18^F-fluorideoxyglucose (^18^FDG), a glucose analogue, are not suitable for long-term monitoring due to their short half-lives. It is necessary to combine two imaging modalities to address this defect. In recent study, an MRI/SPECT/fluorescent tri-modal probe (^125^I-fSiO4@SPIOs) was synthesized by labeling fluorescent silica-coated SPIO with ^125^iodine to quantitatively track MSCs transplanted intracerebrally or intravenously into stroke rats, and the therapeutic efficacy of different injection routes and possible therapeutic mechanisms were evaluated. Neurobehavioral outcomes were significantly improved due to the upregulation of VEGF, basic fibroblast growth factor (bFGF), and tissue inhibitor of matrix metalloproteinase-3 (TIMP-3), although IC-infused MSCs migrated to the lesion site along the corpus callosum and IV-injected MSCs were primarily entrapped in the lung [39].

### 4.3. Fluorophore and Reporter Gene Expression Labeling Techniques

Optical imaging systems incorporating fluorescent imaging (FLI) and bioluminescence imaging (BLI) are used for whole-body imaging but with lower resolution and sensitivity. Fluorescent nanoparticles are suitable for stem cell long-term monitoring [132] and do not affect cell viability and proliferation. Luciferase produces a natural form of chemiluminescence during substrate oxidation. Stem cells transfected with the luciferase reporter gene are detectable using BLI, which is both noninvasive and quantitative. In one study, endothelial colony-forming cells (ECFC) were infected with a lentivirus containing eGFP and fLuc grafted into a photothrombotic (PT) stroke model. Strong BLI signals suggested that ECFCs migrate into the ischemic region [133]; overall, it was possible to monitor endogenous neural stem cells (eNSCs) in a PT stroke model using BLI *in vivo*. The stereotactic injection of conditional lentiviral vectors (Cre-Flex LVs) encoding fLuc and eGFP in the SVZ of nestin-Cre transgenic mice generates specifically labeled eNSCs. This results in significant increases in BLI signals, indicating the proliferation of eNSCs. Additionally, BLI signals relocalize from the SVZ toward the infarct region during the 2 weeks following stroke, demonstrating that nestin-positive eNSCs originating from the SVZ promote proliferation, migration toward the infarct region, and differentiation into both astrocytes and neurons during ischemic stroke [134]. In another study, labeled umbilical cord-derived mesenchymal stem cells (UMSCs) with multigold nanorod (multi-GNR) crystal-seeded magnetic mesoporous silica nanobeads (GRMNBs) were further transfected with lentivirus-luciferase protein (Luc-GRMNBs-UMSC). Photoacoustic PA signals suggested Luc-GRMNBs-UMSC homing to the infarcted area with the aid of a magnet and 7T MRI were suitable for the long-time tracking of transplanted stem cells. MRI revealed multiple low signals located inside the damage site, indicating Luc-GRMNBs-UMSCs migrated to the stroke region [135]. It is necessary to understand the primary distribution and homing of eNSCs *in vivo* because stroke affects neurogenesis in the adult mammalian brain.

Recent studies investigating tracer agents that are currently available for stem cell tracking in stroke are displayed in Table 1.

## 5. Conclusion

Several preclinical and clinical trials have shown that stem cell therapy for cerebral ischemia is safe and feasible and has the ability to promote neurologic functional recovery. However, the precise mechanisms underlying the benefits of stem cell transplantation have not yet been fully elucidated. To achieve optimal therapeutic effects and enhance our understanding of the mechanisms by which stem cells promote functional recovery in neurological disorders, it is essential that we develop noninvasive, reproducible, and quantitative *in vivo* imaging approaches to track stem cell fate. Additionally, the combination of different labeling agents facilitates better and long-term stem cell tracking *in vivo* with appropriate safety and feasibility. Each imaging modality has advantages and disadvantages, and the combined use of different imaging modalities strengthens their respective advantages, allowing us to gain a better understanding of the homing, distribution, and differentiation of implanted cells *in vivo*.

## Figures and Tables

**Table 1:** Tracer agents currently available for tracking stem cells in stroke.

Tracer agent	Imaging modality	Labeled cell type	Route of administration	Results
Radiotracer				
^111^In oxine	SPECT	hUTC	IV	Approximately 1% of transplanted cells migrate to the site of injury, increasing vascular and synaptic densities in the IBZ [38]
Nanoparticles				
SPIOs	3.0T MRI	MSCs	IA	Safe and feasible; ipsilateral MCA conditions and infarction volume affected the number of cells grafted [119]
	4.7T MRI	NSCs	IC	The majority of contralaterally grafted NSCs migrated to the peri-infarct area [76]
	MRI, BLI	hNSCs	IC	Tracking the fate and function of implanted cells in real time for 2 months [41]
MPIOs	MRI	eNSCs/NPCs	IC	Immediate, cell-independent MPIO accumulation at the site of injury [136]
	MRI	hMSCs	IC	Good label stability, did not affect hMSC viability [112]
FMNC	MRI	MSCs	IC	Safe and high efficiency for cell labeling, migration, and accumulation in the ischemic region [118]
fmSIO_4_@SPIONs	3.0T MRI	NPCs	IC/IA	High MR sensitivity and cell labeling efficiency [117]
AIE NPs	FLI	BMSCs	IC	Low cytotoxicity and feasible [137]
GRMNBs	PA, 7.0T MRI, IVIS	MSCs	IV	Enhanced stem cell homing and reduced infarct volume, allowed short- and long-term monitoring [135]
MGIO	1.5T MRI	hfMSCs	IV	Low toxicity and feasible [138]
Gd-DTPA	MRI	BMSCs	IC	Safe and high efficiency [139]
Report gene				
D-luciferin	BLI	BMSC	IP	Higher signal intensity of luciferase-expressing BMSCs 2 h after transplantation and migration to the IBZ [140]
Fluc and eGFP condition lentiviral vectors (Cre-Flex-LVs)	BLI, MRI	eNSCs	IC	A significant increase in eNSC proliferation and migration, and 21% of cells differentiated into astrocytes and neurons [134]
GFP and Luc2 double fusion reporter gene	BLI	ECFC	IA	Functional recovery, improved angiogenesis, neurogenesis, and increased apoptosis [133]

SPECT: single photon emission computed tomography; hUTC: human umbilical tissue-derived cells; IV: intravenous; IBZ: the ischemic boundary zone; SPIOs: superparamagnetic iron oxide; MRI: magnetic resonance imaging; MSCs: mesenchymal stem cells; IA: intra-arterial; MCA: middle cerebral artery; NSCs: endogenous neural stem cells; IC: intracerebral; BLI: bioluminescence imaging; hMSCs: human MSCs; MPIO: micron-sized superparamagnetic iron oxide; eNSCs: endogenous NSCs; NPS: neural progenitor cell; FMNC: fluorescent magnetite nano cluster; fmSIO_4_@SPIONs: fluorescent mesoporous silica-coated superparamagnetic iron oxide nanoparticles; AIE NPs: fluorescent nanoparticles with aggregation-induced emission; FLI: fluorescent imaging; BMSCs: bone marrow-derived MSCs; GRMNBs: multigold nanorod (multiGNR) crystal-seeded magnetic mesoporous silica nanobeads; PAI: photoacoustic imaging; IVIS: interactive video information system; MGIO: microgel iron oxide; hfMSCs: human fetal MSCs; IP: intraperitoneal; Fluc: firefly luciferase; eGFP: enhanced green fluorescent protein; eNSCs: endogenous NSCs; ECFC: endothelial colony-forming cell.
